# Recent advances in *de novo* protein design: Principles, methods, and applications

**DOI:** 10.1016/j.jbc.2021.100558

**Published:** 2021-03-18

**Authors:** Xingjie Pan, Tanja Kortemme

**Affiliations:** 1 Department of Bioengineering and Therapeutic Sciences, University of California San Francisco, San Francisco, California, USA; 2 UC Berkeley – UCSF Graduate Program in Bioengineering, University of California San Francisco, San Francisco, California, USA; 3 Quantitative Biosciences Institute (QBI), University of California San Francisco, San Francisco, California, USA

**Keywords:** *de novo* protein design, computational protein design, protein structure, biophysics, Rosetta, PDB, FASTER, fast and accurate side chain topology and energy refinement, LOCKR, latching orthogonal cage-key proteins, MSD, multistate design, PDB, Protein Data Bank, RIF, rotamer interaction field, SEWING, structure extension with native-substructure graphs, TERMs, tertiary structure motifs, TR-Rosetta, transform-restrained Rosetta

## Abstract

The computational *de novo* protein design is increasingly applied to address a number of key challenges in biomedicine and biological engineering. Successes in expanding applications are driven by advances in design principles and methods over several decades. Here, we review recent innovations in major aspects of the *de novo* protein design and include how these advances were informed by principles of protein architecture and interactions derived from the wealth of structures in the Protein Data Bank. We describe developments in *de novo* generation of designable backbone structures, optimization of sequences, design scoring functions, and the design of the function. The advances not only highlight design goals reachable now but also point to the challenges and opportunities for the future of the field.

The “*de novo*” protein design describes the generation of new proteins with sequences unrelated to those in nature based on physical principles of intramolecular and intermolecular interactions (1). Although most current contributions to the *de novo* design focus on new structures, efforts in the field are increasingly directed toward designing new biological functions and their applications (1, 2). Designer proteins are beginning to impact biomedical and synthetic biology research. Exciting recently designed functions include inhibitors of viral infections (3, 4), immune system modulators (5, 6), self-assembling biomaterials (7, 8, 9), sense-and-respond signaling systems (10, 11, 12, 13), and protein logic gates (14, 15).

Underlying these successful applications are developments of computational design principles over the last decades. Many such principles have been learned from the wealth of existing architectures in the Protein Data Bank (PDB) (16). While many computational design applications modify existing proteins (12, 17, 18, 19, 20), it is becoming possible to design both structures and functions entirely *de novo* (1). It was recognized early that variations of helical architectures could be designed based on parametric equations (21). Helical bundle proteins have indeed proven to be very “designable” (22) and have consequently been adapted to many functions (13, 14, 15, 23, 24, 25, 26, 27). More recent developments have expanded the structural repertoire of *de novo* proteins to other fold classes (28, 29, 30, 31, 32). The first new alpha-beta protein, with a fold not previously observed in nature, was assembled from fragments from the PDB (33). Subsequent careful analyses of natural protein architectures led to the design of different alpha-beta proteins (30), including a symmetrical artificial TIM barrel (34), and all-beta proteins (29, 31).

Toward new functions, recent computational advances have led to the ability to generate precise geometric variations in *de novo*–designed protein families, mimicking the ability of evolution to precisely tune the shapes of the members of protein families for new activities (28, 32). Although these designed proteins are not close in sequence to any naturally occurring proteins, principles from structures in the PDB are still the guiding design. Such principles are useful for generating new protein structures through assembly from continuous (33, 35) or discontinuous (25, 36, 37) three-dimensional elements, as well as for the development (38) and optimization (39, 40) of design energy functions used to rank design candidates. Moreover, the most recent developments of deep learning for protein structure prediction (41, 42, 43) foreshadow new methods in the design, taking advantage of learned principles of the protein structure (44, 45).

Computational methods have addressed a number of key challenges in the protein design and will continue to play a major role in advancing applications. Computational protein design is typically defined as at optimization problem: given a user-defined structure and function, find one or a few low-energy amino acid sequences stably adopting the desired structure and performing the targeted function. Ongoing challenges for designing *de novo* functional proteins arise from all major aspects of this process (Fig. 1): generation of designable protein backbone conformations, sampling of sequences optimal for these structures, scoring functions that are sufficiently accurate to distinguish correct from incorrect solutions, and design of functional sites with the desired activities. In this review, we discuss development of design principles and methods in these aspects and will highlight the role played by the structural data in the PDB in informing these principles, in the context of this special issue of the *Journal of Biological Chemistry* celebrating the 50th anniversary of the PDB. We focus on advances made in the past 5 years. For readers interested in the history of *de novo* protein design, we refer to a recent review (46).

**Figure 1:**
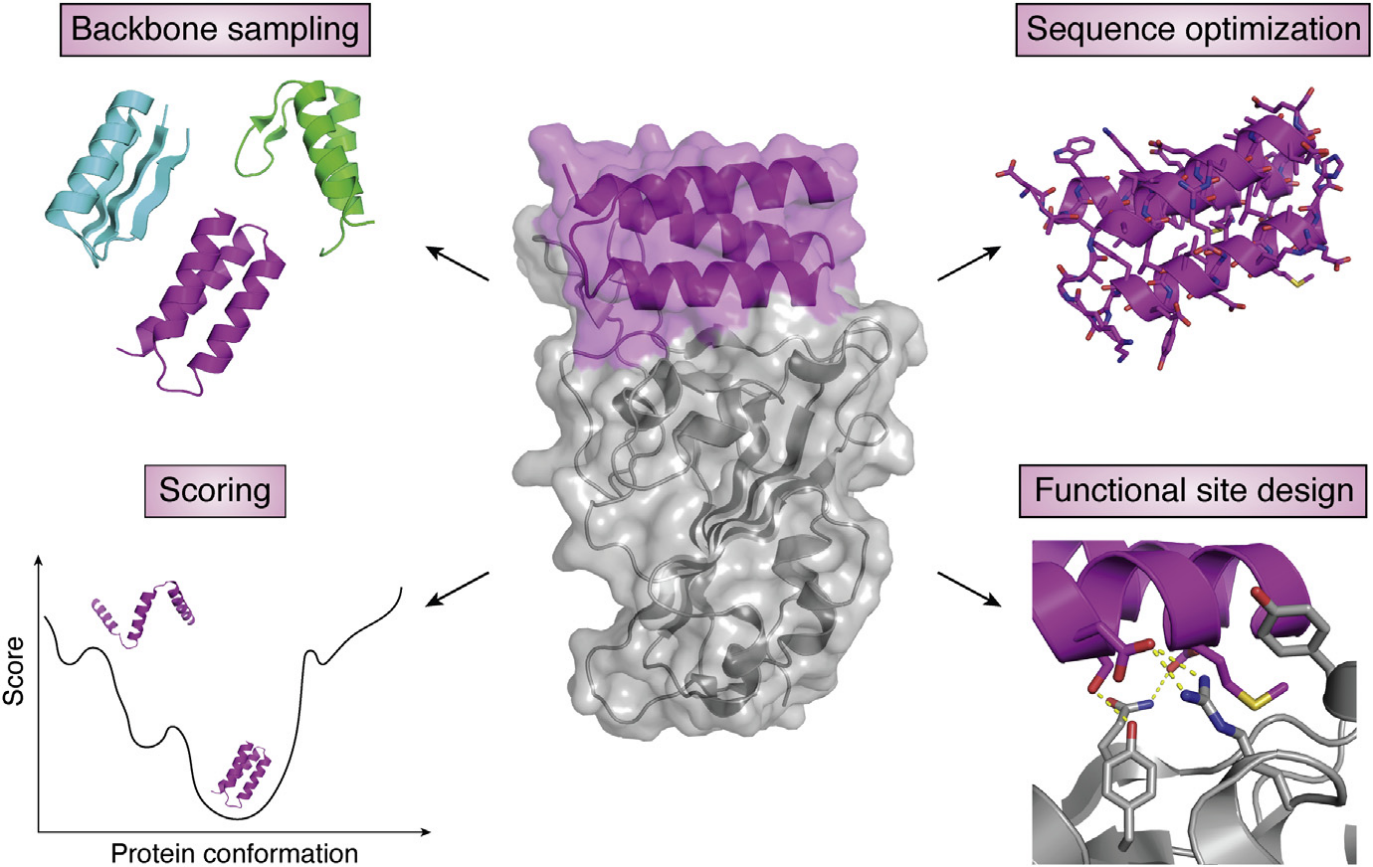
**Major aspects of the *de novo* protein design.** The design of a functional *de novo* protein, for example, a binder (*middle*, *magenta*) to a target protein (*middle*, *gray*), requires sampling of the backbone structure space to find a backbone compatible with the function, sequence optimization to stabilize the backbone, and designing the functional site interactions. A scoring function is necessary to select designs with desired properties, typically by identifying low-energy sequence–structure combinations.

## Sampling of *de novo* backbone structures for the protein design

Backbone structures determine the overall shapes of proteins and therefore play a critical role in protein functions. Even small proteins (100 residues or less) have hundreds of backbone degrees of freedom, making it impossible to sample the backbone structure space by brute force. Moreover, because folded proteins need to have well-packed cores and satisfied hydrogen bonds, only a small fraction of the backbone structure space can stably exist, that is, is “designable” (47, 48). In the following sections, we describe different levels of sampling backbone conformations for the design, starting from variation of existing structures and ranging to the design of novel folds, fold families, and constrained peptides, and ending with a perspective on the backbone design by emerging machine learning methods.

### Variation of existing structures

A workaround to the difficulty of *de novo* backbone design is redesigning native backbone structures from the PDB for new functions (18, 19, 20). Because proteins are not static, state-of-the-art design methods typically consider small structural adjustments in response to sequence changes, or to diversify native backbones. In particular, several approaches have been developed to mimic “back-rub” motions (49, 50), a common mechanism for interconverting between alternate backbone conformations observed in high-resolution (≤1 Å) crystal structures (51). A back-rub motion involves internal backbone rotations about axes between C-alpha atoms. Incorporating such back-rub moves into design simulations has led to considerable improvements in modeling structural changes in point mutants (49, 50, 52), protein dynamics on fast timescales (53, 54), prediction of molecular recognition specificity (55), and the sequence design (56).

### Helical bundles

Helical bundles were the first type of protein fold designed *de novo* at atomic accuracy (22, 57). Owing to their regularity, backbone structures of coiled-coil helical bundles can be sampled near exhaustively by Crick’s parameterization (21). The availability of a method to systematically sample helical bundle backbones and the high stability (58) of the fold make helical bundles a good model system for designing a broad scope of functions such as ligand binding (25), ion transport (24), and switches (15). More details on recent progress of the coiled-coil design can be found in a review by Woolfson (59).

### *De novo* design by assembling local structures

**Figure 2:**
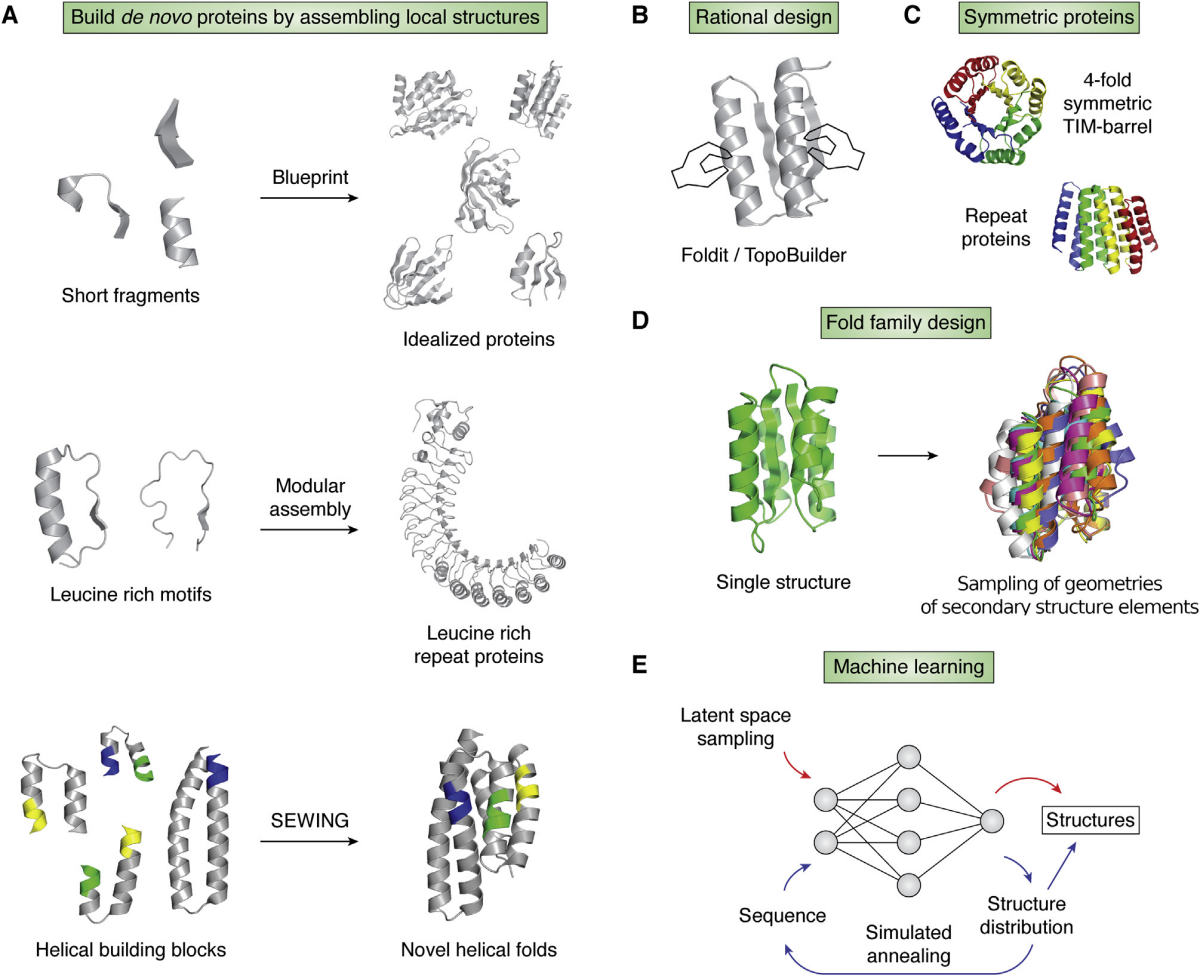
**Advances in *de novo* backbone generations**. *A*, methods to build *de novo* proteins by assembling local structures. The blueprint method assembles fragments of three or nine residues into idealized structures with different fold topologies (29, 30, 31, 33, 61, 62, 64). Modular leucine-rich motifs are connected into repeat proteins with defined curvatures (65). The SEWING method (36) connects local structural elements into helical proteins with novel folds. Overlapping regions are colored. *B*, the Foldit game (71) and TopoBuilder (72) let players or experts rationally design the atomic details of backbone structures. *C*, symmetry reduces the complexity of backbone generation. Symmetry was used to design a 4-fold (*colors*) symmetric TIM barrel (34) and repeat proteins (67). *D*, *de novo* protein fold families can be generated by sampling the geometries (length, as well as relative position and orientation) of secondary structure elements (28, 32). *E*, generative machine learning methods (*red*) build novel backbone structures by latent space sampling (81). The hallucination method (45) (*red*) uses the TR-Rosetta neural network to predict the structure distribution of a sequence. The sequence is optimized using Monte Carlo–simulated annealing by maximizing the divergence between the predicted structure distribution and a background distribution representing unstructured proteins. SEWING, structure extension with native-substructure graphs; TR, transform-restrained.

*De novo* backbones beyond helical bundles can be designed by a fragment assembly strategy originally used in structure prediction (35, 60). Typically, the first step in design is defining a blueprint that specifies the lengths and relative orientations of secondary structure elements. Short fragments with desired secondary structures are then extracted from the PDB and assembled into a three-dimensional protein model (Fig. 2*A*). Top7 was the first protein designed by this method and has a fold topology not observed in nature (33).

The blueprint strategy was subsequently generalized to design *de novo* backbones for a number of different fold topologies. Notably, each fold topology required specific design rules derived from native structures in the PDB. For instance, idealized alpha-beta fold proteins favor certain β-hairpin chirality, relative orientations of alpha-beta and beta-alpha units, and ranges for the values of backbone torsion angles in the connecting loops (30, 61). Proteins with curved β-sheets need bulges and register shifts to enable defined β-sheet curvatures (62). The jelly roll fold topology is constrained by loop conformations, side-chain directionality, and β-strand length (31). β-Barrel proteins require glycine kinks and β-bulges to reduce Lennard–Jones repulsive interactions (29). Traditionally, *de novo*–designed proteins were validated using low throughput assays. Recent developments in large-scale DNA synthesis (63) now enable high-throughput stability screening of *de novo*–designed small proteins (64). A recent screen identified thousands of sequences encoding stable designs with four different target structures and identified features of the models associated with design success.

Other strategies for *de novo* backbone generation do not use blueprints but still use assembly of protein fragments borrowed from nature. Proteins with controllable curvatures can be designed by combinations of modular leucine-rich-repeat units (65) (Fig. 2*A*). The structure extension with native-substructure graphs (SEWING) method (36) combines continuous or discontinuous helical building blocks from existing proteins (Fig. 2*A*). SEWING first extracts small substructures from proteins in the PDB. Substructures that share high similarity in local regions are overlapped and combined. Finally, loops are designed to close the gaps between discontinuous elements. Notably, previous applications of Crick’s parameterization to the design were restricted to the coiled-coil topology, while SEWING allows the exploration of more diverse helical topologies.

A recent method called AbDesign (66) seeks to mimic natural homologous recombination. In contrast to other methods, Abdesign uses larger segments and relies on the similarity between members of the same protein family to facilitate backbone sampling. In particular, AbDesign breaks proteins from a structure family into a few modular segments based on structural alignments and then recombines these segments into new backbones. AbDesign is able to build large numbers of similar structures even for moderately sized families of homologs.

The complexity of the backbone design problem can be reduced by symmetry (Fig. 2*C*). A 4-fold symmetric TIM barrel was designed using the blueprint fragment assembly strategy described above (34). Experimental characterization of the designs revealed important hydrogen bonds defining the strand register between repeat units. Tandem repeat proteins made of a series of identical helix–loop–helix–loop structural motifs can be systematically assembled (67). The designed repeat proteins span a broad range of curvatures. By modulating the curvature, alpha tandem repeat proteins can form closed toroid structures (68). A large number of proteins with diverse shapes can be generated by designing rigid junctions to connect helical repeat proteins (69).

### Backbone design by fragment assembly using human intuition

Human rationale can design the atomic details of *de novo* proteins (Fig. 2*B*). The developers of the online game Foldit (70) crowd-sourced solutions for the challenge of *de novo* protein design (71). Online Foldit players were provided with a set of tools to generate, mutate, move, and score protein structures. Starting from a fully extended peptide chain, players were able to fold the chain into *de novo* structures and stabilize the structures by sequence optimization. The players designed more than ten million models. The Foldit developers experimentally tested 146 top designs and identified 56 designs that adopted well-folded monomeric structures. The experimentally solved structures of four of these designs closely agreed with the computational models.

A different strategy incorporates human expert knowledge into the process of backbone generation for design. The TopoBuilder (72) protocol lets designers build proteins in a bottom-up approach starting from functional motifs (*e.g.*, a helix in a binding interface). Designers define the sizes and three-dimensional coordinates of secondary structure elements. The coordinates are then transformed into constraints for the Rosetta FunFolDes (73) method to build all-atom models. The TopoBuilder protocol successfully designed protein binders (72).

### Fold family design

Naturally occurring proteins with the same fold topology can have distinct functions because of fine-tuned differences in the precise geometries of structural elements (74, 75). The ability to explore such geometric variation within fold families is critical for design of new protein functions that require precise three-dimensional conformations of active sites. The recently developed loop-helix-loop unit combinatorial sampling method systematically samples loop-helix-loop geometries in arbitrary protein folds by near exhaustive testing of combinations of short loops (32) (Fig. 2*D*). The generated protein geometries had similar distributions to those observed in native structures in the PDB but also included thousands of new structures. Experimentally solved structures spanned a wide range of the sampled distribution. Using a different approach to geometric variation, an enumerative algorithm was developed to sample diverse pocket structures of nuclear transport factor 2 fold proteins (28). Parameters such as sheet curvatures, loop types, and secondary structure lengths were sampled during a hierarchical backbone assembly process. Thousands of stable designs with diverse pocket geometries were identified by a high-throughput yeast surface display experiment.

### Constrained peptides

Naturally occurring constrained peptides can have strong pharmacological activities. The GenKIC method (76) adapted the robotics-inspired kinematic closure algorithm (77, 78) from loop modeling, generalized the approach to sample noncanonical backbone degrees of freedom, and applied it to cyclic peptides and peptides constrained by disulfide bonds. The designed peptides closely matched the experimentally solved structures and showed high stability against thermal and chemical denaturation. Kinematic closure methods in Rosetta (76, 78) can be used to enumerate backbones of cyclic peptides with seven to ten residues nearly exhaustively (79). GenKIC was also applied to design meso-size proteins stabilized by multivalent cross-linkers (80).

### Backbone design by machine learning

Machine learning models trained with the rich structural data from the PDB are able to generate novel protein backbone structures (Fig. 2*E*). A generative adversarial network (81) model builds protein structures represented as pairwise distances between all backbone atoms. A pretrained deep convolutional neural network then recovers the three-dimensional backbone structure from pairwise distances. Some of the designed structures could be recapitulated by fragment-based structure prediction methods (82). Another variational autoencoder–based model focused on generating immunoglobulin structures (83). The model learned the distribution of immunoglobulin structures and compressed the distribution into a low-dimensional space termed latent space. Immunoglobulins with defined complementarity determining regions can then be generated through latent space sampling. A new method used the idea of neural network “hallucination” (generation of structures) for the protein design (45). The model repurposes the neural network from transform-restrained (TR)-Rosetta (42). The TR-Rosetta network is a fast method to predict the inter-residue contact map of an arbitrary sequence. A loss function is defined as Kullback-Leibler divergence (84) between the TR-Rosetta neural network–predicted contact map and a background distribution. Novel sequences and structures can be designed simultaneously by optimizing the loss function through Monte Carlo–simulated annealing. Diverse structures were designed by the model and shown to be folded by experimental characterization.

## Sequence optimization

After generation of protein backbones, the second step in a typical *de novo* protein design protocol is selection of amino acid side-chain types and conformations to stabilize the backbone conformation and to adopt specific three-dimensional active site geometries optimized for function. Early *de novo* design studies used amino acids that favor specific secondary structure types (85) or binary polar/hydrophobic patterns (86) to define protein structures. Because side-chain conformations are clustered as rotamers (87, 88), the side-chain design can be formulated as a discrete optimization problem (89), that is, find a combination of rotamers that minimize the energy of a structure. The complexity of the problem grows exponentially with the increase of the number of residues. Small-scale side-chain design problems can be solved deterministically by the dead-end elimination algorithm (90), but many *de novo* protein side-chain optimization problems are too large to be solved deterministically. Instead, amino acid sequences and side-chain conformations are often optimized using Monte Carlo methods (91, 92), which do not guarantee to find the global minimum, but the solutions are often sufficiently accurate for applications.

**Figure 3:**
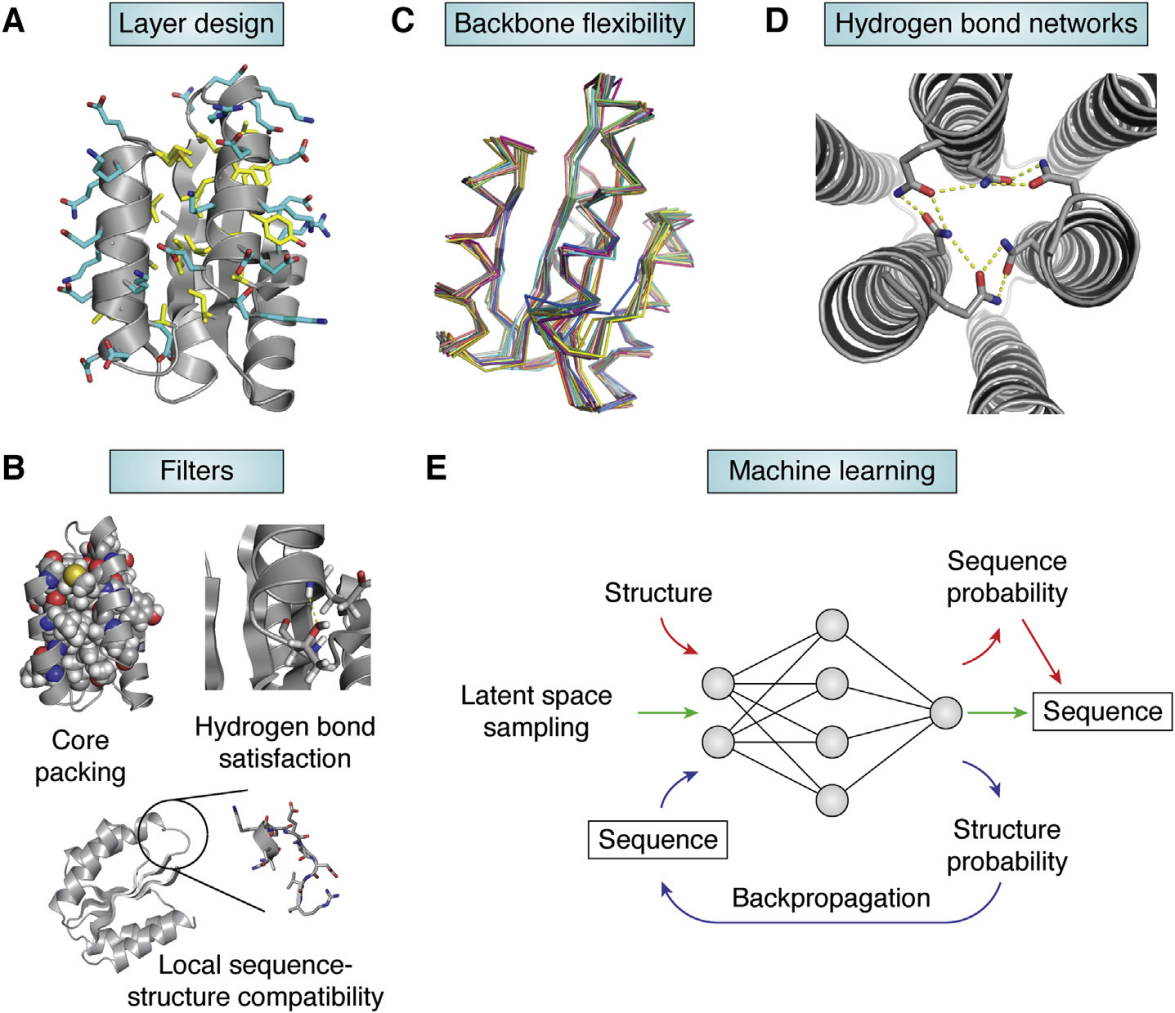
**Advances in side-chain design.***A*, in layer design, polar residues (*cyan*) are only allowed at surface and boundary positions, while hydrophobic residues (*yellow*) are only allowed at boundary and core positions. *B*, structures generated by side chain design methods can be evaluated by a set of filters, such as core packing quality, hydrogen bond satisfaction and local sequence/structure compatibility. *C*, side chain design methods that exploit backbone flexibility outperform fixed backbone methods (98). *D*, the HBNet method (100) designs hydrogen bond networks. *E*, neural networks can predict the probabilities of sequences given a backbone structure (102, 103) (*red*). Generative machine learning models design sequences by latent space sampling (104, 105, 106, 107, 108) (*green*). The TR-Rosetta neural network predicts the probability of the structure of a given sequence. The difference between the desired structure and the predicted structure can be backpropagated through the neural network to optimize the sequence (109) (*blue*). TR-Rosetta, transform-restrained Rosetta.

The efficiency of side chain sampling methods can be improved by constraining the amino acid types allowed at each residue position. LayerDesign is a common strategy (17, 31, 32, 62, 64) to constrain designable amino acid types (Fig. 3*A*). Residue positions are divided into three categories: core, boundary, and surface. The core region allows only hydrophobic amino acids, the surface region allows only polar amino acids, and the boundary region allows all amino acids. The LayerDesign method increases sampling speed and reduces artifacts, such as buried polar residues, which may result from insufficient sampling or scoring errors. To further eliminate flawed designs, the results from Monte Carlo samplers are often filtered by a set of properties such as core packing (93) and hydrogen bond satisfaction (32) (Fig. 3*B*). A high-throughput stability screen of designed small proteins showed that buried nonpolar surface area and local sequence-structure compatibility had strong correlations with the stabilities of designs (64).

### Sequence optimization with flexible backbones

Solutions of fixed backbone side-chain design problems are sensitive to the backbone structures used as input. Because the Lennard-Jones potential term in scoring functions (see the section below) scales as the 12th power of distance when two atoms are close to each other, a small adjustment to the backbone structure may result in a considerable energy change. To address these problems, state-of-the-art side-chain design methods sample both side-chain rotamers and local backbone conformations (50, 52, 94, 95) (Fig. 3*C*). Typically, methods that exploit backbone flexibility or use backbone ensembles outperform the fixed backbone design (96, 97). A study benchmarked (98) several flexible backbone side-chain design methods including CoupledMoves (94), BackrubEnsemble (56), and FastDesign compared with a fixed backbone design method using the same scoring function. Methods that simultaneously, rather than sequentially, optimize sequence and backbone structure, such as CoupledMoves (94), may be advantageous (98).

### Hydrogen-bonding networks

Hydrogen bonds play an important role in the specificity of protein–ligand and protein–protein interactions. The formation of a hydrogen bond only allows narrow ranges of distances and orientations between the donor and acceptor groups (38). Almost all hydrogen bond donor or acceptor groups in a protein must form hydrogen bonds within the protein or with solvent molecules to avoid large energetic penalties of unsatisfied hydrogen bonds (99). The HBNet method addresses the challenges for the hydrogen bond design by systematically searching for possible hydrogen-bond networks (100) (Fig. 3*D*). HBNet constructs a graph whose nodes are rotamers that have hydrogen bond donors or acceptors. Two nodes are connected by an edge if the rotamers of the nodes can form hydrogen bonds. Hydrogen bond networks can be generated by traversing the graph. HBNet was successfully applied to design helical bundle homo-oligomers with specificity mediated by hydrogen bond networks. A Monte Carlo version of the HBNet method uses a stochastic algorithm to traverse the HBNet graph (101). This new approach significantly improves the sampling speed and makes larger design problems possible.

### Sequence design using machine learning methods

A number of machine learning methods for protein sequence design were developed recently (Fig. 3*E*). Deep neural network methods were trained to predict probabilities of amino acids at each residue position of a backbone structure (102, 103). Generative models learn distributions of protein sequences and can generate new native-like protein sequences with or without input backbone structures. A number of generative models were developed for sequence design, including generative adversarial networks (104), variational autoencoders (105, 106), and graph-based (107, 108) models. Notably, the structure prediction neural network from TR-Rosetta (42) can be repurposed for sequence optimization (109). For a protein sequence, the TR-Rosetta neural network predicts distances, angles, and dihedrals for every pair of residues. A loss function is defined as the difference between the prediction and the target structure. The gradient of the loss is then back-propagated through the TR-Rosetta neural network to optimize the sequence. Combining machine learning models and traditional Monte Carlo samplers improves performance over every single method (103, 109).

## Scoring functions for the design

Scoring functions in the computational protein design aim to distinguish designs with desired properties from those not adopting the intended structures and functions, typically by identifying low-energy sequence–structure combinations. Early protein energy functions (110) used harmonic terms for bond energies and a Lennard–Jones potential for van der Waals interactions. Modern physics–based energy functions (111, 112, 113) account for additional energy terms such as electrostatics and desolvation. An alternative approach to physics-based energy terms is using statistics from known structures to derive potential functions (114). The first version of the scoring function in the Rosetta program for structural modeling, and the design was developed for protein structure prediction (115) and was a statistical potential function derived from structures in the PDB (16, 116) using Bayesian statistics (35). To adapt Rosetta for the protein design, all-atom detail and physics-based terms were incorporated (33, 38), which in turn led to considerable advances in both protein structure prediction and protein design (82, 117). The current version of the Rosetta force field used for design is similar to modern molecular mechanics force fields (40, 118), but including orientation dependency of hydrogen-bonding interactions based on PDB statistics and electronic structure calculations (38, 119); the orientation dependence of hydrogen bonding is important for designing interaction specificity critical to many functions (14, 100, 120). In the following, we highlight recent developments in scoring functions for membrane proteins and for interactions with nonprotein molecules, as well as scoring approaches that learn from structures in the PDB.

### Membrane scoring functions

**Figure 4:**
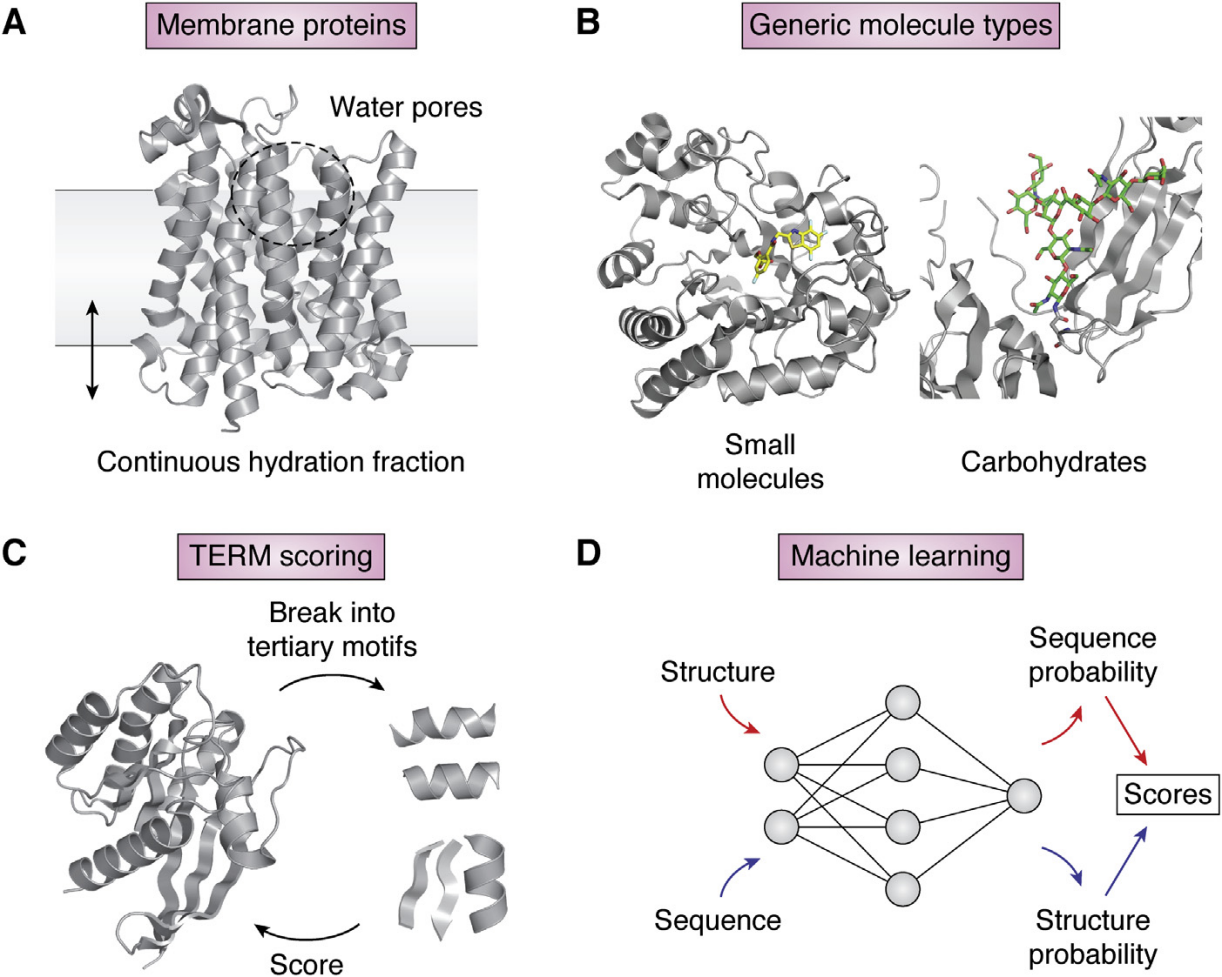
**Advances in scoring functions**. *A*, a membrane scoring function (124) uses a continuous hydration fraction to calculate the free energy change of residues from water to the lipid environment. Water pores in membrane proteins are explicitly modeled. *B*, protein design scoring functions are generalized to model small molecules (132) and carbohydrates (131). *C*, the TERMs-based scoring function (133) breaks proteins into tertiary structure motifs and evaluates the fitness of the sequence for any local structure using the sequence profiles of the tertiary motifs. *D*, machine learning methods predict the probability of sequences given a structure (102) or the probability of structures given a sequence (109). The predicted probabilities can be used as scores for the compatibility between sequences and structures. TERMs, tertiary structural motifs.

Scoring functions for soluble proteins take advantage of the large number of solved structures in the PDB to validate and fit the parameters of the score function (121, 122). Transmembrane proteins make up about 30% of ORFs in known genomes but are currently underrepresented in the PDB, complicating the development of membrane protein scoring functions. An early version of the Rosetta membrane scoring function (123) used statistics from 28 transmembrane proteins to fit parameters and was validated by *ab initio* structure prediction of 12 multipass membrane proteins. Recently, a new membrane scoring model (124) was developed, which aims to better capture the heterogeneous membrane environment (Fig. 4*A*). The interface between bulk water and bulk lipid is modeled as a continuous transition of hydration fraction, with water-filled pores modeled using a convex-hull algorithm (125). The water-to-bilayer transfer energy is then calculated using the hydration fraction and the Moon and Fleming hydrophobicity scale (126). This membrane model improves performance in several computational tests, including prediction of membrane protein orientation, calculation of changes in membrane protein stability upon mutation, discrimination of native structures from incorrect models, and the extent to which the native sequence is recovered in design simulations.

### Scoring interactions with nonprotein molecules

Many protein functions involve interactions with other types of molecules such as DNA, RNA, saccharides, or small molecules. Expanding the types of molecules supported by scoring functions is critical for designing such protein functions. Scoring functions for DNA (127) and RNA (128) have been successfully applied to structure prediction and design (129, 130). Recently, a scoring function was developed for saccharide and glycoconjugate structures (131) (Fig. 4*B*). Benchmarking results on docking problems showed that the scoring function has the ability to predict binding of glycan ligands. Small molecules have highly diverse combinations of chemical groups, making it challenging to transfer parameters calculated for representative molecules to other molecules. A new approach (132) simultaneously optimized all parameters in a small-molecule energy function guided by thousands of small-molecule crystal structures. The resulting scoring functions significantly improved docking success rate.

### TERM-based scoring

Protein design methods typically seek to find low-energy sequences for a given target structure, but this approach does not consider if there are alternative structures a sequence can adopt that have even lower free energies. One way to overcome this limitation is by directly calculating the fitness for a given structure in the protein sequence space. Protein structures can be broken up into three-dimensional local pieces called tertiary structural motifs (TERMs) (133) (Fig. 4*C*). Half of the structures in the PDB can be described by only about 600 TERMs (37), indicating that the sequence preferences of each TERM could be used to calculate the fitness of a sequence for a given local structure. A strong correlation (133) was observed between the TERM-derived scores and protein structure model accuracies from the Critical Assessment of Structure Prediction. Recently, the TERM score was used to predict protein–peptide binding energies and design peptide binders of antiapoptotic proteins Bfl-1 and Mcl-1 (134).

### Protein scoring functions by machine learning methods

The power of machine learning models to learn the statistical representations underlying rich sequence and structural data provides new perspectives for protein structure prediction and design (41, 42, 44, 135) (Fig. 4*D*). Neural network models trained with evolutionary sequence data and structures from the PDB outperform traditional methods in structure prediction (41, 42, 135). Most recently, it has been proposed that neural networks that predict inter-residue orientations (defined by three dihedral and two planar angles) can be inverted for assessing the probability of the desired structure for a given sequence; in principle, such an approach could be used as a scoring function for protein design to evaluate the fitness of a sequence across an entire structural landscape (109). Another approach using a deep convolutional neural network scoring function seeks to predict the probability distribution of amino acid types at each residue position conditioned on the local environment (102).

## Design of new protein functions

Proteins perform functions by placing atoms with certain physicochemical properties at specific positions in the three-dimensional space. Initial work on the functional protein design directly borrowed from native functional site “motifs” (three-dimensional arrangements of functional groups in an existing active site) (136). Recent developments and successful applications of *de novo* protein structure design methods are gradually overcoming the limitations imposed by the use of existing functional sites, beginning to make it possible to both design the precise placement of arbitrary functional groups and the protein environment *de novo*. In the following sections, we describe advances in the design in the areas of binding proteins for ligands and other proteins, large protein assemblies, membrane proteins, and protein switches.

### Ligand-binding sites

Ligand binding is a common function for native proteins. The *de novo* ligand-binding site design requires high accuracy in sampling and scoring. Specificity of ligand binding is often realized by polar interactions which are highly sensitive to the positions and orientations of polar groups. A misaligned hydrogen bond could cause a considerable free energy penalty and reduce the binding affinity by an order of magnitude. Early studies designed *de novo* binding sites by manually defining side chains that form favorable interactions with ligands (11, 20, 26). An effort that uses HBNet and a Monte Carlo sequence design algorithm to design hydrogen bonds resulted in designs that bind to ligands, but a crystal structure revealed that the ligand is rotated 180° in the pocket around a pseudo-two-fold axis in the compound (137). The authors suggested that the sampling methods failed to model subtle structural changes and that the scoring function underestimated desolvation energies for the ligand. This result highlights the challenges inherent in sampling and energy evaluation in binding-site designs.

**Figure 5:**
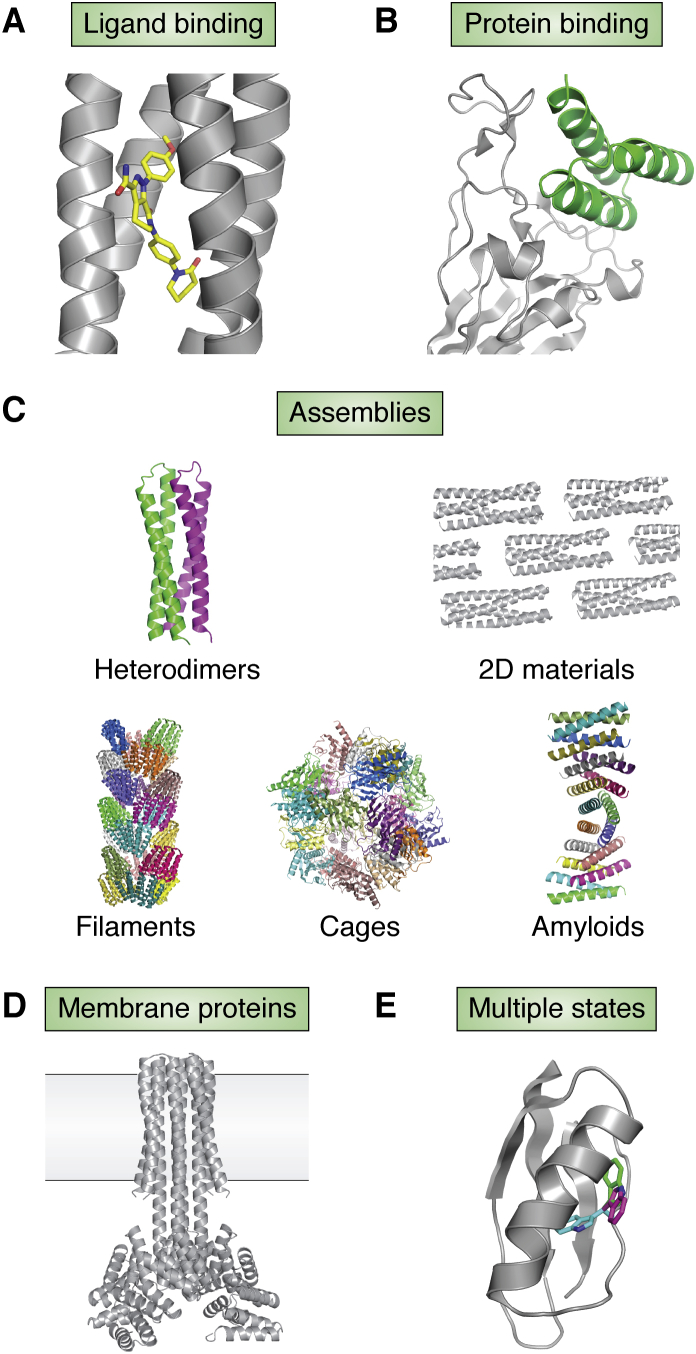
**Advances in design of new protein functions.***A*, a apixaban (*yellow*) binder designed by the Convergent Motifs for Binding Sites (COMBS) algorithm (25). *B*, A *de novo* protein (*green*) binds the severe acute respiratory syndrome coronavirus 2 (SARS-CoV-2) spike protein (*gray*) (4). *C*, *de novo* proteins self-assemble into heterodimers (120), two-dimensional materials (9), filaments (8), cages (140), and alpha amyloids (143). *D*, a *de novo*–designed multipass transmembrane protein that has a defined membrane orientation (148). E. the designed DANCER protein has a tryptophan side chain that switches between predicted conformational states on the millisecond timescale (152).

Recent developments in binding site–generation methods aim to address these challenges. The rotamer interaction field (RIF) docking method (29) generates an ensemble of billions of discrete amino acid side chains that make hydrogen-bonding and hydrophobic interactions with the target ligand. The method then searches for protein backbone scaffolds that are able to present ligand-binding side chains with the appropriate geometry. RIF docking was successfully applied to design a binding site for the fluorogenic compound DFHBI into a *de novo* beta barrel scaffold (29). Two other methods use the structural information in the PDB to generate binding-site ensembles (25, 138). These methods break the ligand into smaller substructures (fragments) and find protein residues that interact with the ligand fragments from the PDB. The interacting residues are combined into binding sites by Monte Carlo–simulated annealing (138) or built onto backbone scaffolds by an algorithm called Convergent Motifs for Binding Sites (25). The Convergent Motifs for Binding Sites method was applied to engineer *de novo* proteins that bind the drug apixaban with low and submicromolar affinity (Fig. 5*A*).

### Protein binders

Similar to the ligand binding–site design, designing protein binders to target proteins requires high accuracy scoring and sampling. A workaround to these challenges is using binding motifs from known protein–protein interfaces. Proteins that bind to influenza hemagglutinin and botulinum neurotoxin B (3) were designed by building known helical motifs that bind to the intended targets onto *de novo* designed small protein scaffolds (64). Several hundred high-affinity binders were validated by a high-throughput yeast surface display assay. Likewise, proteins that bind to the interleukin-2 and interleukin-15 receptors were designed by building a helical bundle from interface helices of native interleukin-2 and interleukin-15 (6).

Although difficult, interaction interfaces can also be designed without native motifs. Recently, the RIF docking method originally developed for the small-molecule binding site design was applied to design small helical bundle proteins that bind to the severe acute respiratory syndrome coronavirus 2 (SARS-CoV-2) spike protein (4) (Fig. 5*B*), yielding binders with affinities ranging from high nanomolar to micromolar. After experimental optimization, the most potent design had a 100-pM affinity to spike.

### Protein assembly

Several design studies have addressed the problem of the protein–protein interface design where both sides of each interface are designed, leading to protein assembly (Fig. 5*C*). Homo-oligomers with cyclic symmetries were designed by systematic enumeration of arrangements of the monomers followed by the interface design (139). A set of heterodimers that have orthogonal binding specificities were designed using parametric backbone generation and HBNet (120). The orthogonal heterodimers can be used to design protein logic gates (14). Self-assembled nanocages with higher-order symmetries were designed by symmetric docking followed by Monte Carlo interface sequence design (140, 141). Fusing the designed cages to membrane binding and endosomal sorting recruiting peptides induced the formation of nanocage-containing extracellular vesicles (142). The strategy of combining symmetric arrangement of protein chains and Monte Carlo interface sequence design was also successfully applied to design protein filaments (8), alpha amyloid-like structures (143), or two-dimensional materials (7, 9).

### Membrane proteins

Proteins that localize to phospholipid bilayer membranes have been designed since the emergence of the *de novo* protein design (144, 145). Membrane-spanning peptides that self-assemble into helical bundles were designed to perform functions such as cofactor binding (146) and ion transport (24). Recent advances have expanded the scope of the membrane protein design. A study of the driving forces of membrane protein stability showed that steric packing of nonpolar side chains alone is sufficient for the folding of membrane proteins (147). Using a steric packing code derived from the natural protein phospholamban, the authors were able to design a synthetic membrane protein stabilized entirely by nonpolar side chains. Accurate multipass transmembrane proteins were designed (148) using a recently developed framework for membrane protein modeling (149) (Fig. 5*D*). Parametrically generated backbones were stabilized by hydrogen bond networks designed with HBNet and Monte Carlo side-chain optimization. Orientations of the designs were specified by incorporating a ring of amphipathic aromatic residues at the lipid-water boundary on the extracellular side and a ring of positively charged residues on the cytoplasmic side. This strategy was then applied to design transmembrane pores (150). Although there was no explicit modeling of ligands that can pass through the pores, several designs displayed ligand specificity: a designed 12-helix pore selectively passed potassium over sodium, and a designed 16-helix pore (but not the 12-helix pore) enabled the passage of biotinylated Alexa Fluor 488.

### Conformational changes

Among the most challenging functions to design are conformational changes between multiple states. A single-state design would be successful as long as the designed state resides in a deep energy minimum, so that sizable scoring errors can often be tolerated (151). However, the multistate design (MSD) requires considerable accuracy in scoring relative stabilities, such that the probability distributions among multiple states can be modeled correctly. In addition, the MSD must simultaneously optimize several objectives, for example, the energies of each state and the energy differences between states. This multiple-objective optimization problem adds significant challenges to the sequence design. A recently developed meta-MSD protocol designed a protein that has a tryptophan side-chain switching between defined conformational states on the millisecond timescale (152) (Fig. 5*E*). Meta-MSD used a back-rub ensemble of backbones (56) as the input. Side chains were then designed by optimizing the Boltzmann-weighted average energy of all members from the ensemble using the fast and accurate side chain topology and energy refinement algorithm (153). The energy landscape of a designed sequence was estimated using energies of each backbone structure from the ensemble. Sequences with energy landscapes that supported desired conformational dynamics were selected as final designs.

### Protein switches

**Figure 6:**
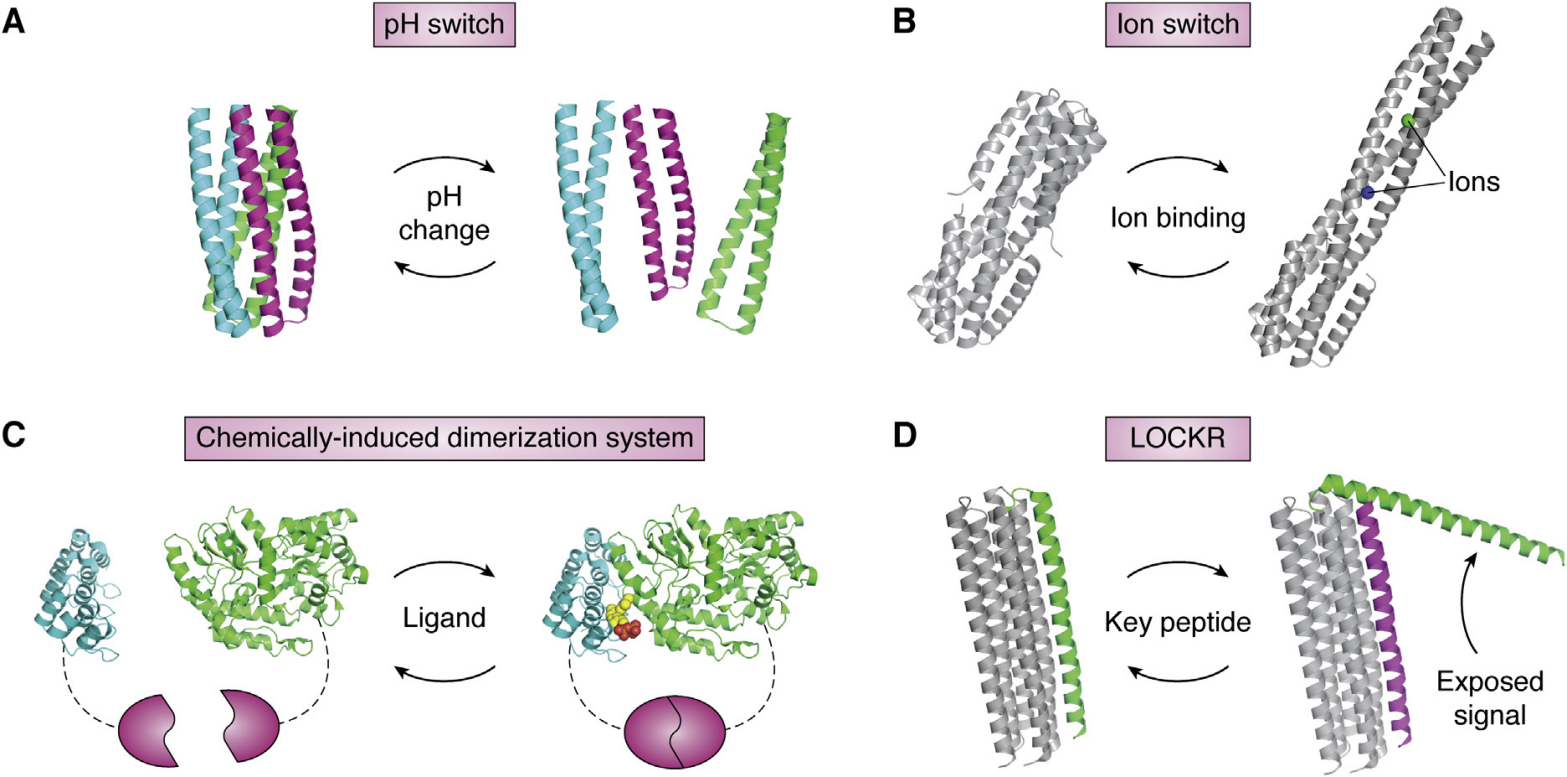
**Advances in the design of protein switches that change conformation in response to diverse signals.***A*, a designed helical trimer changes its oligomerization state in response to pH changes (155). *B*, a designed helical bundle protein changes conformation upon binding to a calcium ion (*green*) and a chloride ion (*blue*) (156). *C*, a designed artificial chemically induced dimerization system (12) assembles upon binding to a farnesyl pyrophosphate ligand (*spheres*), linking ligand binding (sensing) to a modular response through reconstitution of a split output module (*gray*, *magenta*). *D*, in the LOCKR system, a helical peptide “key” (*magenta*) can displace and expose a signal peptide (*green*) (15). LOCKR, latching orthogonal cage-key proteins.

Protein switches change their conformations when triggered by external signals, adding a potential extra layer of complexity over designing proteins that adopt multiple conformations. However, designing switches could be seen as a more tractable problem because the external trigger can introduce a large free-energy bias toward one state, making the design success less sensitive to scoring errors. An early study described a protein designed to switch between two distinct target folds triggered by the addition of Zn^2+^ (154). The authors used a Monte Carlo side-chain design method to optimize the sum of energies of the two folded states, showing that it is possible to design protein switches by solving a single-objective optimization problem. Following similar principles, other proteins were designed to change the oligomerization state in response to a pH change (155) (Fig. 6*A*) or change conformations in the presence of Ca^2+^ (156) (Fig. 6*B*). A modular protein switch that senses a small molecule was designed through an induced dimerization mechanism (12) (Fig. 6*C*). A ligand binding site for farnesyl pyrophosphate was designed *de novo* at the interface of a protein–protein heterodimer complex. The designed proteins dimerized in the presence of the farnesyl pyrophosphate ligand and were able to transduce several modular downstream signals such as the enzyme activity, fluorescence, or luminescence. Latching orthogonal cage-key proteins is another recently designed protein switch system (15), consistent of a helical bundle and a helical peptide called key (Fig. 6*D*). The key peptide can displace a helix in the bundle and expose a signal on the displaced helix. The latching orthogonal cage-key proteins system was used to induce protein degradation and localization (15), target cells with precise combinations of surface antigens (23), and detect viral proteins (13).

## Future perspectives

**Figure 7:**
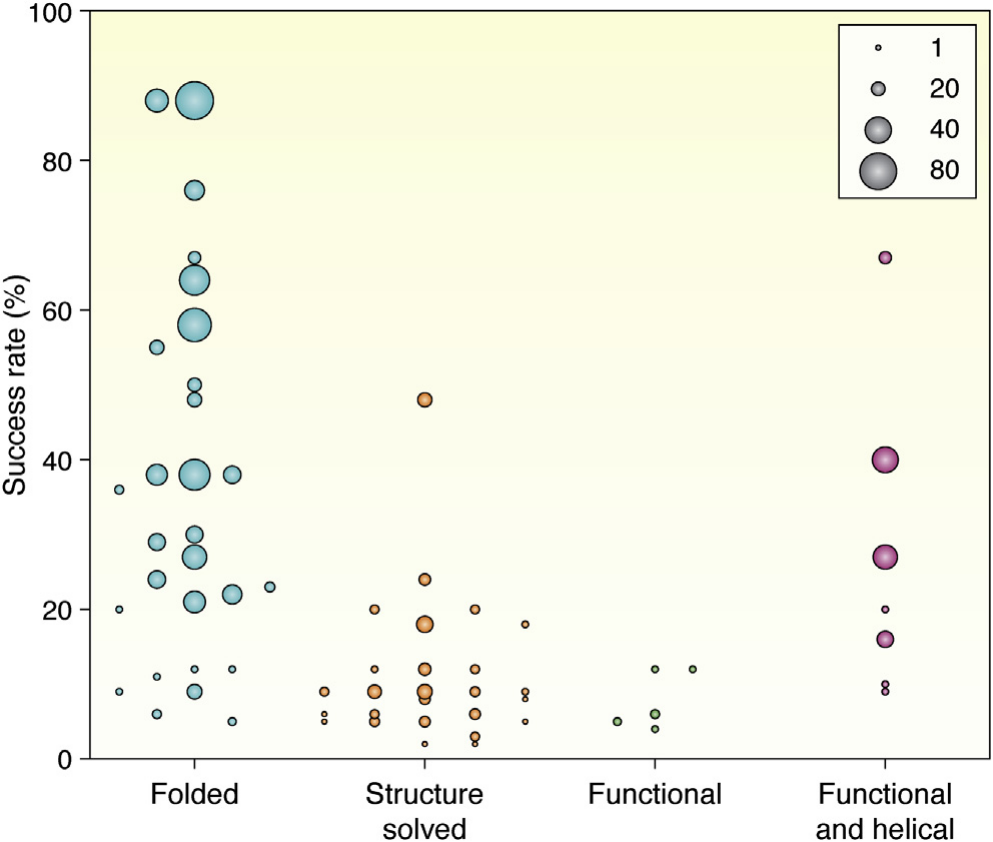
**Success rates reported for design studies listed in**Table 1**.** The success rate is defined as the percentage of reported designs in each study that adopt the designed structure (*folded*, *blue*; experimental structure determined, *orange*) or function (*green*, *red*). The *circle* size denotes the number of folded/functional designs in each study. The success rates for studies where proteins were *de novo*–designed to have new structures are varied but can be high with many designs (*blue*). In contrast, success rates and numbers of successful designs for proteins with new functions (*green*) are much lower, except in a few cases where functional designs were all-helical proteins (*red*). Only studies that reported ten or more experimentally characterized designs (Table 1) are included. “Folded” refers to designs that were characterized by CD and/or NMR spectroscopy or had an experimentally determined structure, displayed the expected oligomerization state (if measured), and/or were functional (if designed to have a function).

The development of computational methods for *de novo* protein design in the last two decades has expanded the scope of designable protein structures and functions considerably. Automatic computational tools have enabled nonexperts to accurately design well-folded *de novo* proteins (71). However, the *de novo* protein design is not a solved problem. Because proteins have highly diverse structures and functions, the difficulties of design problems also have great variations (Fig. 7, Tables 1 and 2). While robust protocols exist for designing helical bundles and small, idealized proteins with certain alpha-beta fold topologies (30, 58, 64), the success rates for other proteins such as beta barrels can be low (29, 31, 34). Addressing those challenging problems still requires significant amount of expertise, and sometimes trial and error. Challenges are particularly apparent in the design of proteins with new functions (Fig. 7). New protein structures can be designed with considerable success rates without experimental optimization (Table 1), but the activities of proteins derived directly from the computational design are often weaker than achievable activities of naturally evolved proteins. Therefore, computational designs are often (although not always) optimized by experimental methods such as site saturation mutagenesis (4, 20).

**Table 1:** Success rates of designs tested by low- to medium-throughput experiments

Design goal and reference	Designs tested	Soluble	Folded (CD)	Correct monomer/oligomer	Folded (NMR)	Solved structure	Functional^a^
Highly stable helical bundles (58)	9	5	5	5		3	
Ideal α-β proteins (30)	54	45	32	17	16	5	
Ideal α-β proteins (61)	72	64	47	39	17	6	
Proteins with curved β-sheets (62)	66	58	53	54	25	8	
Proteins with the jelly roll topology (31)	19	16	2	2	2	1	
Novel helical folds (36)	11	8	4	4		2	
FoldIt player designed proteins (71)	146	101	56	66		4	
4-Fold symmetric TIM barrels (34)	22	22	5			1	
Leucine-repeat proteins (65)	29	29	25	22		7	
Repeat proteins (67)	83	74	72	53		15	
Repeat proteins with closed toroid structures (68)	20	10				4	
*De novo* fold families (32)	45	24	17	17	17	4	
Constrained peptides (76)	137					12	
Peptide macrocycles (79)	23				11	11	
Design by deep network hallucination (45)	129	129	27	32			
Helical bundles with hydrogen bond networks (100)	114	101	101	66		10	
Fentanyl binding proteins (11)	62					1	3
Digoxigenin binding proteins (20)	17					2	2
Porphyrin binding protein (26)	1	1	1	1	1	1	1
Apixaban binding proteins (25)	6	6	6			1	2
Fluorescence-activating β barrels (29)	56	38	16	22		1	2
IL-2 and IL-15 mimics (6)	12					1	8
Repeat proteins using rigid helical junctions (69)	34	33	33	30		4	28
Cyclic protein homo-oligomers (139)	96	64		21		5	15
Orthogonal protein heterodimers (120)	97	94		85		6	39
60-Subunit protein dodecahedron (141)	17	3		2		1	2
Protein filaments (8)	124	b				6	34
α Amyloid peptides (143)	6	b	6			4	4
Two-dimensional protein arrays (7)	62	b				4	4
Two-dimensional protein arrays (9)	10	b					2
Zn^2+^ transporter (24)	1	1		1	1	1	1
Multipass transmembrane proteins (148)	7	b	6	6		2	6
Transmembrane pores (150)	23^c^	17	2	3		2	2
Multistate proteins (150)	4	4	4	4	4	1	3
pH-triggered switches (155)	5	5	5	4		2	4
Metal ion–triggered switches (156)	20	20	15	11		4	2
LOCKR protein system (15)	1	1	1	1			1
Split biosensors (12)	9					1	2

LOCKR, latching orthogonal cage-key proteins.

a Here we use a broad definition of functions, including, for example, membrane localization or formation of defined complex structure.

b Successful designs can be insoluble.

c Designed soluble proteins were converted into transmembrane proteins.

**Table 2:** Success rates of designs tested by medium- to high-throughput experiments

Design goal and reference	Round	Designs screened	Stable designs	Designs with designed functions	Success rate (%)
Mini-proteins (64)	1	3560	206		6
	2	2984	231		8
	3	4154	496		12
	4	3980	1855		47
NTF2 fold family (28)	1	2709	578		21
	2	5188	1499		29
Influenza hemagglutinin binders (3)	1	7276		40	0.5
Botulinum neurotoxin B binders (3)	1	3406		874	26
*De novo* SARS-CoV-2 miniprotein inhibitors (4)	1	100,000		105	0.1
Epitope presenting proteins (72)	1	10^6^–10^8^		201,684^a^	0.2–20

SARS-CoV-2, severe acute respiratory syndrome coronavirus 2.

a Stability and binding were selected together.

There are many areas in the field of the computational *de novo* protein design where significant progress is needed. To make large sequence optimization problems computationally tractable, scoring functions use a number of approximations such as implicit solvation models and pairwise decomposable energy terms. Improving scoring accuracy and speed will continue to be an important direction. Current backbone geometry sampling methods are limited to certain secondary structures and fold topologies. Developing methods that expand the space of designable backbones will greatly expand reachable functions. Although a variety of *de novo* protein functions have been designed, most functions cannot be designed routinely. Methodological advances are needed to design the intricate geometries of protein functional sites with increasing precision, such that subsequent experimental optimization can be minimal. Such developments are particularly important for fine-tuned and controllable conformational changes, and highly polar functional sites. Applying design protocols on different problems and testing the methods systematically can be valuable for identifying and addressing limitations. Emerging machine learning methods provide opportunities and challenges in this relatively new subfield. Machine learning methods can not only synthesize existing data into statistical models that generate novel proteins but also iteratively integrate experimental data to guide the protein design (157). The best design strategies for many problems might be combinations of machine learning models and advances in existing design methods. Recent advances in designing basic functions including ligand binding, protein–protein interaction, membrane localization, and induced switching are making it possible to envision the design of more complex and composite functions such as artificial cellular signaling systems, motors, and controllable molecular machines built from elementary components designed *de novo*.

## Conflict of interest

The authors declare that they have no conflicts of interest with the contents of this article.
